# Modelling floppy iris syndrome and the impact of pupil size and ring devices on iris displacement

**DOI:** 10.1038/s41433-020-0782-7

**Published:** 2020-02-04

**Authors:** David Lockington, Zhaokun Wang, Nan Qi, Boris Malyugin, Li Cai, Chenglei Wang, Hui Tang, Kanna Ramaesh, Xiaoyu Luo

**Affiliations:** 1 Tennent Institute of Ophthalmology, Gartnavel General Hospital, Glasgow, UK; 2 Department of Mechanical Engineering, Hong Kong Polytechnic University, Hong Kong, China; 3 Institute of Marine Science and Technology, Shandong University, Shandong, China; 4 S. Fyodorov Eye Microsurgery Federal State Institution, Moscow, Russia; 5 School of Mathematics and Statistics, Northwestern Polytechnical University, Xi’an, China; 6 School of Mathematics and Statistics, University of Glasgow, Glasgow, UK

**Keywords:** Lens diseases, Technology

## Abstract

**Introduction:**

The aim of this paper was to further develop a previously described finite element model which equates clinical iris billowing movements with mechanical buckling behaviour, simulating floppy iris syndrome. We wished to evaluate the impact of pupil dilation and mechanical devices on normal iris and floppy iris models.

**Methods:**

Theoretical mathematical modelling and computer simulations were used to assess billowing/buckling patterns of the iris under loading pressures for the undilated and dilated normal iris, the undilated and dilated floppy iris, and additionally with a mechanical ring device.

**Results:**

For the normal iris, billowing/buckling occurred at a critical pressure of 19.92 mmHg for 5 mm pupil size, which increased to 28.00 mmHg (40.56%) with a 7 mm pupil. The Malyugin ring device significantly increased critical initiating buckling pressures in the normal iris scenario, to 34.58 mmHg (73.59%) for 7 mm ring with boundary conditions I (BC I) and 34.51 mmHg (73.24%) with BC II. For the most floppy iris modelling (40% degradation), initiating buckling value was 18.04 mmHg (−9.44%), which increased to 28.39 mmHg (42.52%) with the 7 mm ring. These results were much greater than for normal undilated iris without restrictive mechanical expansion (19.92 mmHg).

**Conclusion:**

This simulation demonstrates that pupil expansion devices inhibit iris billowing even in the setting of floppy iris syndrome. Our work also provides a model to further investigate the impact of pupil size or pharmacological interventions on anterior segment conditions affected by iris position.

## Introduction

Ophthalmic surgeons require good pupillary dilation to effectively address the surgical challenges of modern cataract surgery. Intracameral mydriatic preparations are increasingly utilised to avoid complications arising from insufficient pupil size in ocular surgery [1–6]. Such adjuncts can include a bolus injection of a pharmacological agent to cause pupillary dilatation (such as phenylephrine), or as part of the constant irrigation fluidics during phacoemusification to limit pupillary constriction (such as adrenaline) [1–6]. However, there are still occasions where the use of a mechanical device is indicated [7–9]. Femtosecond laser cataract surgery has been associated with the release of prostaglandins into the aqueous humour and subsequent pupillary constriction and it has been reported that up to 10% of cases of inadequate pupillary dilatation will still need mechanical pupil expansion, using adjuncts such as iris hooks or ring devices [10–12]. One such device is the popular Malyugin ring (MicroSurgical Technology Inc., Redmond, Washington, USA), which was launched in 2007. This square foldable polypropylene ring device was based on the original loop IOL design of Fyodorov. It has four circular scrolls located at equidistant points on the ring to allow eight iris-retaining points of attachment, thus stabilising the central pupil margin and limiting abnormal iris movement, with the second version providing a fixed 7 mm pupil opening [13–15].

In 2005, Chang and Campbell originally described the association of systemic use of the α-1A antagonist tamsulosin and intraoperative floppy iris syndrome (IFIS), an adverse surgical situation which they clinically described as iris miosis, billowing and a tendency for iris prolapse [16]. Mechanical pupil devices such as the Malyugin ring have been shown clinically to successfully restrict iris movement in IFIS, and prevent iris prolapse [17]. Intracameral phenylephrine has also been successfully used as a pharmacological method to address IFIS, but there have been some concerns raised regarding the off-label use of this surgical adjunct [18]. The advent of commercial intracameral products containing phenylephrine, such as Mydrane (Thea, Clermont), should alleviate these concerns regarding accuracy of dosage [1, 5, 18].

We have previously modelled floppy iris syndrome and described the mechanical engineering concept of iris buckling (billowing) via a mathematical computer simulation [19]. Fundamental mechanics show that a thin plate structure will move (buckle) into a wavy mode under a critical external force, and this principle allows us to understand and evaluate the potential mechanics involved in iris movement. This is the engineering principle behind the billowing patterns originally clinically described in IFIS [16]. These modes of buckling can be induced at lower critical pressures dependent on iris parameters, such as a lower Young’s modulus, as in floppy iris syndrome. We subsequently modelled the impact of the stiffening agent of intracameral phenylephrine on iris movement, and noted that its effect was enhanced in the model due to pupillary dilation [20]. To further our understanding of this clinical scenario, we wished to model and simulate the impact of a restrictive pupil device on iris movement, both in the setting of normal iris parameters, and in floppy iris syndrome.

## Methods

In this study, the finite element method was used to simulate iris movement through buckling behaviour (billowing shapes), with and without the Malyugin ring. The detailed parameters for our mathematical model can be found in the published literature [20]. Firstly, we considered the stability of a reduced iris structure subject to uniform loading pressure increments and obtained its buckling modes and corresponding critical pressures as a baseline reference. Secondly, we applied a dilated inner diameter of 7 mm and associated boundary conditions (BC) to mimic the restrictive Malyugin ring effect on the pupil, and compared the iris’ new buckling modes and corresponding critical loading pressures. Thirdly, through reducing the parameters for the elastic properties of the iris, we also investigated the effectiveness of the 7 mm Malyugin ring on limiting iris movement/buckling in the modelled setting of floppy iris syndrome.

### Specifics of modelling

#### Iris geometry and three-dimensional modelling

In our numerical model, the iris was modelled as an axisymmetric annular disc with a central aperture. According to previously published data, we assumed that a normal iris had a uniform thickness of *T*_I_ = 0.34 mm, the pupil diameter was *D*_*P*_ = 5 mm and the diameter of the iris external edge was 11 mm [20]. A 7 mm dilated pupil diameter was used to mimic the addition of the Malyugin ring. Assuming volume conservation (due to material incompressibility), the thickness of the iris model was 0.3904 mm with the 7 mm Malyugin ring. A cylindrical coordinate system was adopted for the analysis, with its origin located at the centre of the outer circle of the iris model, and its radial direction *R*, azimuthal direction *θ* and longitudinal direction *Z* defined as illustrated in Supplementary Fig. A.

#### Iris properties

The material of the iris was assumed to be linear elastic and homogeneously orthotropic. To assist with the modelling, a Poisson’s ratio of 0.499 was applied to ensure the iris was incompressible. Furthermore, the azimuthal elastic modulus *E*_*θ*_ and radial elastic modulus *E*_*r*_ were set to be 2.97 kPa, and 4.00 kPa, respectively. The other material properties, such as longitudinal modulus *E*_*z*_ and the shear modulus *G*_*rz*_, *G*_*θz*_ and *G*_*rθ*_, were determined using the previously described methodology [20].

#### Boundary and loading conditions

As the iris is anatomically attached to the ciliary body (i.e. secured at the peripheral boundary), the outer edge of the iris model was assumed to be fixed in all our simulations.

### Testing scenarios

#### Initiation of iris buckling in normal iris (undilated and dilated)

In the first instance, assessment of the normal iris and subsequent buckling patterns following incremental loading pressures in the setting of undilated and dilated pupils was conducted without the inclusion of pupil expansion devices. The inner edge of the iris model was set free. A 10 mmHg base pressure was simultaneously applied on the upper and lower surfaces of the iris as well as the inner edge, which was then gradually increased with a constant increment of 1 mmHg to evaluate the potential buckling of the iris model. This method of testing, in conjunction with the iris properties, boundary and loading conditions provide a reduced model of the iris shape as a circular disc, secured peripherally with a central opening and enables comparison of size and magnitude of iris displacement under various testing conditions.

#### Initiation of iris buckling in normal iris (dilated by mechanical Malyugin ring)

In the second set of testing, the eigenvalue buckling analysis was performed to evaluate the effectiveness of the 7 mm Malyugin ring on the subsequent iris buckling. The loading conditions were the same as that in the first set. The effect of the Malyugin ring was realised using two different BC. boundary conditions I (BC I) involved pinning all three degrees of freedom at eight uniformly distributed contact points with the iris inner margin, as shown in Supplementary Fig. B. Secondly, with boundary conditions II (BC II) the pupil margin was pinned down in only two degrees of freedom (*r*, *z*) at the four points of contact where the iris was engaged in the helical scrolls of the Malyugin ring device. This secondary BC modelling was intended to simulate more realistically the potential clinical behaviour of a floppy iris which typically is more elastic in nature and can enlarge circumferentially. At the other four points where the iris only wraps around the straight limbs of the device the model iris was pinned down in only one degree of freedom (*r*).

Following initial simulations and consultation with clinicians involved in this project, this reduced modelling was felt to be most consistent with the restrictive clinical nature of the ring device and the various limitations and assumptions of the iris simulation.

#### Initiation of iris buckling/billowing in floppy iris scenario without mechanical restriction, and with dilation by mechanical Malyugin ring

In the third set of testing, we simulated the buckling of floppy iris behaviour without mechanical restriction, and then finally with the addition of the Malyugin ring. The floppy iris properties were represented by reducing the iris elastic parameters, including Young’s modulus. The boundary and loading conditions were the same as those for the normal iris with the Malyugin ring (2nd set of testing parameters). The simulation was conducted with different material stiffness, where the reduction of elastic moduli, *E*_*i*_ (*i* = *r*, *θ*, *z*), was realised by multiplying a proportion factor *η*. In this study, two proportion factors (*η* = 0.6 and 0.8) were considered.

## Results

For convenience of discussion, the bucking mode number ‘*n*’ was defined according to the number of local maxima across the iris structure in our simulations [20]. Table 1 details the initial critical buckling pressure for all the simulations, and details the results (as a percentage increase ratio) to the 5 mm undilated normal iris to provide a context for comparison of magnitude for iris billowing behaviour.

**Table 1:** Comparison of the critical pressures to initiate iris buckling of different iris models with different material parameters, detailing the true value and the increased percentage ratio when compared with the normal undilated 5 mm iris.

Iris model	Iris initial critical buckling pressure					
	*η* *=* 0.6 (More floppy)		*η* *=* 0.8 (Mild floppy)		*η* *=* 1.0 (normal)	
	Value (mmHg)	Increase ratio (%)	Value (mmHg)	Increase ratio (%)	Value (mmHg)	Increase ratio (%)
With undilated pupil (diameter 5 mm)	18.04	−9.44	18.99	−4.67	19.92	–
With pupil dilated to 7 mm	23.86	19.78	25.92	30.12	28.00	40.56
With 7 mm M-ring (BC I)	28.39	42.52	31.49	58.08	34.58	73.59
With 7 mm M-ring (BC II)	28.27	41.92	31.39	57.58	34.51	73.24

The increase ratio ‘*R*’ can be calculated by using the following equation:

$$R = \frac{{P \, - \, P_N}}{{P_N}} \times 100\% ,$$

where ‘*P*’ is the iris initial critical buckling pressure in different cases, and ‘*P*_*N*_*’* is the initial critical buckling pressure of the normal undilated 5 mm iris model.

*BC* boundary conditions.

### Initiation of buckling of normal iris with undilated or dilated pupil

**Fig. 1:**
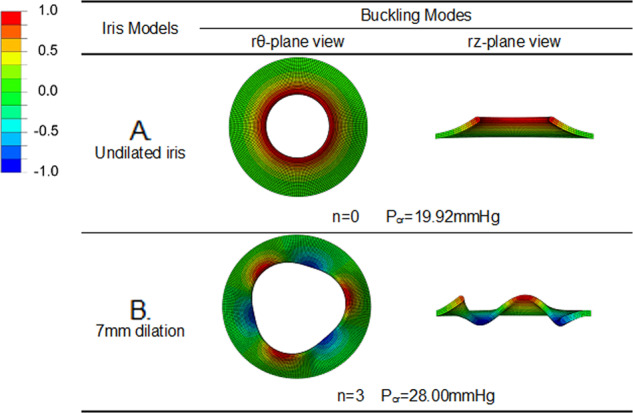
The critical buckling values for normal iris. **a** The undilated normal iris model; **b** the iris model with pupil expansion 7 mm; in which ‘*n*’ is the buckling mode and Pcr is the critical buckling pressure. The colour bar indicates the mode’s longitudinal displacement. Note that the magnitude is unified in the eigenvalue analysis.

The first occurring buckling mode of the normal iris with the undilated pupil (diameter 5 mm) and dilated pupil (7 mm) illustrates that the required critical pressures to cause iris buckling increases with increasing pupil dilation. Specifically, buckling occurred at a critical pressure of 19.92 mmHg for the undilated iris, whereas it occurred at 28.00 mmHg with the pupil dilated to 7 mm (40.56% increased ratio) (see Fig. 1).

### Inhibition of iris buckling in normal iris (dilated by mechanical Malyugin ring)

**Fig. 2:**
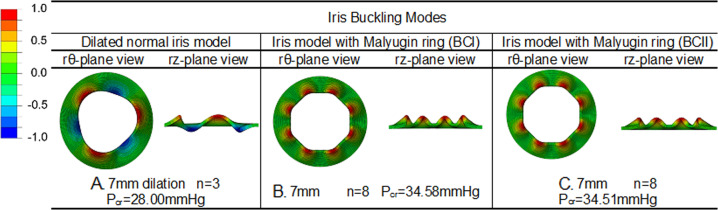
The initiating buckling values for the normal dilated iris model compared with the 7 mm Malyugin ring model. **a** Normal iris model with pupil dilation 7 mm. **b** Normal iris model with 7 mm Malyugin ring using boundary conditions I (BC I). **c** Normal iris model with 7 mm Malyugin ring using BC II.

The addition of the Malyugin ring restriction on the dilated normal iris model, simulated using both BC I and BC II, greatly increased the critical pressure required to initiate iris buckling. Interestingly, the two different BCs gave very similar results. The critical initiating pressures for iris buckling with the 7 mm Malyugin ring was 34.58 mmHg for BC I and 34.51 mmHg for BC II (compared with the similarly dilated normal iris without a device value of 28.00 mmHg) (Fig. 2).

#### Initiation of iris buckling/billowing of floppy iris (without mechanical restriction)

**Fig. 3:**
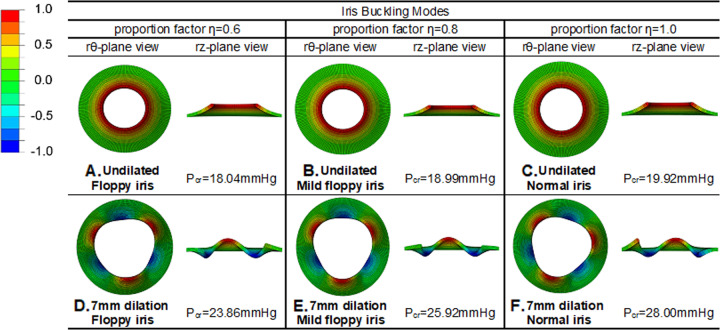
The buckling modes for the dilated iris with different proportion factors 0.6 (floppy iris), 0.8 (mild floppy iris) and 1.0 (normal iris stiffness): **a**–**c** are the buckling modes for undilated 5 mm iris models. **d**–**f** are the buckling modes for pupil dilation 7 mm.

To further investigate iris buckling behaviour in floppy iris syndrome, we repeated the buckling analysis for the iris without the mechanical restriction of the Malyugin ring but having different pupil dilatations and less material stiffness parameters. Two proportion factors, i.e. *η* = 0.6 and 0.8, were applied to reduce the material stiffness. We observed that the critical pressure required to initiate buckling reduced from 19.92 mmHg for the normal iris (*η* = 1.0) to 18.99 mmHg for the iris with *η* = 0.8 and furthermore to 18.04 mmHg for the iris with *η* = 0.6. These results had a negative ratio when compared with the normal undilated results, so demonstrating that a floppy iris is easier to displace than a normal iris (as observed clinically in IFIS) (Fig. 3).

#### Inhibition of buckling/billowing for floppy iris by using Malyugin ring

**Fig. 4:**
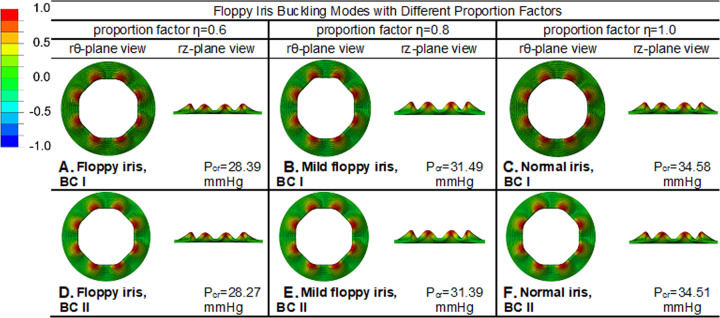
The buckling behaviour for the iris model with the 7 mm Malyugin ring and different proportion factors 0.6 (floppy iris), 0.8 (mild floppy iris) and 1.0 (normal iris stiffness): **a**–**c** are the buckling modes with BC I; **d**–**f** are the buckling modes with BC II.

To investigate the inhibition of iris displacement in the setting of floppy iris by using mechanical devices, the impact of the 7 mm Malyugin ring on floppy iris buckling was analysed considering different proportion factors (*η* = 0.6, 0.8 and 1.0) at the two different types of BC (BC I and BC II), as shown in Fig. 4. Compared with the normal iris simulation results, the Malyugin ring was able to significantly raise the critical pressure required for the floppy irises to initiate buckling patterns, and so stabilise the iris. For example, the critical pressure despite modelling the most floppy iris (*η* = 0.6, equating to a 40% degradation of normal iris stiffness) with a 7 mm Malyugin ring showed a 19.0% increase from 23.86 mmHg to 28.39 mmHg for BC I, and 18.5% from 23.86 mmHg to 28.27 mmHg for BC II.

## Discussion

There are a limited number of published studies analysing the behaviour and relationships of the normal iris in the anterior chamber, and even less which attempt to model various complex cataract surgery scenarios [21–26]. The dynamic coupled characteristics of the iris and the aqueous humour flow has been previously analysed by Heys et al., and presented as a mathematical model to predict the iris deformation mechanics (with aqueous humour modelled as a Newtonian fluid and the iris modelled as a linear elastic solid) [27]. The intrinsic properties of the iris have been previously evaluated from animal data, with a reported iris elastic modulus ranging from 0.88 kPa (porcine data) to 6.2 kPa (bovine data) [28, 29]. A recent study of stress analysis of iris tissue induced by pupil expansion devices used an average elastic modulus of 3 kPa and a Poisson’s ration of 0.49 in their 3D geometric evaluation [30]. This modelling included the Malyugin device, but in order to maintain consistency in their comparison of stress forces on the iris with the other devices (iris hooks and APX), they displaced the four scroll locations in the Malyugin ring modelling by 1.5 mm. They reported that the induced stress on the iris sphincter tissue in their simulation was less traumatic with the Malyugin ring than these other devices, as more uniform circular expansion generated to smallest stress gradients [30]. However, they did not consider other situations involving altered iris parameters, such as floppy iris syndrome.

Our present study aimed to study the potential for abnormal iris displacement (iris buckling) during intraocular surgery, and to consider the effectiveness of the Malyugin ring towards inhibiting these unwanted behaviours. The Malyugin ring was shown to stabilise the iris movement and inhibit iris buckling in our modelling. Our mathematical and computer simulation models have demonstrated that the critical buckling pressures of the iris are dependent on the intrinsic parameters of the iris (undilated versus dilated) and the inner boundary stability (normal versus restricted due to a mechanical device such as the Malyugin ring). [See Table 1 for comparison of magnitude of force for initiation of iris displacement.]

### Clinical relevance of this work

Our study findings indicate that an intrinsically normal iris with sufficient pupil dilatation can effectively inhibit iris buckling, and this corresponds with our clinical experience during routine cataract surgery. When the iris parameters were reduced to simulate IFIS, iris buckling/billowing was shown to be initiated at a much lower value compared with the scenario with normal iris properties. We have shown that as the proportion factor *η* decreases (as in floppy iris), the critical buckling pressure of the iris also decreases, so increasing the possibility of intraoperative iris displacement. A floppy iris requires much less pressure to buckle, and this trend was demonstrated even when modelling different pupil dilatation sizes. To summarise, regardless of how much the floppy iris was dilated, the critical pressure required to initiate displacement was much less than that for normal iris properties. However, when the Malyugin ring was applied to this floppy iris model with even 40% degradation in its elastic moduli, the critical pressures required to initiate the buckling increased significantly. This is consistent with the clinical experience of cataract surgeons when the Malyugin ring device is used to inhibit floppy iris behaviour intraoperatively, where the iris can billow, but not buckle, and confirms the clinical relevance of our modelling [13, 17, 20]. In addition, the magnitude of the buckling should be less with the restrictive ring device, and the pupil margin should not be able to displace sufficiently to cause iris prolapse through a limbal wound. [See online Supplementary video demonstrating extensive iris displacement in the floppy iris scenario compared with reduced iris movement despite higher buckling pressures with Malyugin ring model.]

### Limitations of this model

Our model has certain limitations and assumptions, and cannot model all the abnormal iris movements in floppy iris syndrome, but it would follow that early use of such devices should inhibit iris movement and prevent the extremes of buckling displacement which would lend itself via a pressure gradient to result in iris prolapse through the surgical wounds. We acknowledge our finite element iris model is significantly reduced in its parameters, but this was necessary to make it clinically relevant when testing a theoretical yet realistic IFIS scenario.

In real life, the human iris has been demonstrated by spectral domain OCT to be variable in iris diameter and volume when comparing inferior with superior, and temporal to nasal, but this degree of detail was felt to be beyond the scope of our mathematical model to evaluate floppy iris behaviour [31]. Clinically, deployment of the Malyugin ring in the small pupil scenario (often observed in IFIS) can cause localised iris trauma at the pupil margin which has recently been described in a spatially varying stress pattern model of the iris stroma [30].

This study shows mathematically and through computer simulation that mechanical expansion devices inhibit the onset of floppy iris behaviour but do not eliminate it totally. These concepts were demonstrated regardless of the size of the ring device. Our model does not account for the fact that a larger Malyugin ring can throw the iris into more circumferential folds compared with a smaller ring, as we predominantly focused on the pupil margin. It is believed that pupil expansion rings reduce the floppiness or buckling of the iris in IFIS by restricting the movement of the pupil margin and adjacent iris due to direct contact resulting in a dampening effect. This pinning down of the pupil margin in some degrees of freedom plays a role in reducing the floppiness of the iris rather than the specific size of the ring. For this reason, pupil expansion rings are more effective than pharmacological agents in inhibiting floppy iris behaviour.

## Conclusion

We have shown that greater pupil dilation will increase the critical pressure required to initiate iris displacement, and so act to stabilise the normal iris in routine intraocular surgery. In the setting of floppy iris syndrome, this critical buckling initiation pressure significantly decreases, and the abnormal iris will displace/buckle with limited resistance, leading to the potential of iris prolapse. This study demonstrates via mathematical modelling and computer simulation that the pupil expansion devices are a definitive method to mechanically stabilise abnormal iris behaviour in cataract surgery due to mechanical dilation significantly inhibiting iris buckling (billowing) even in the setting of IFIS. Our work also provides a model for floppy iris syndrome, and would be useful to further investigate the impact of pupil dilation or pharmacological interventions on anterior segment conditions affected by iris position.

## Summary

### What was known

- Floppy iris syndrome can result in abnormal iris displacement in a buckling configuration (billowing behaviour).
- Pupil expansion devices such as the Malyugin ring can restrict floppy iris behaviour.
- There are no other mathematical models or simulations of floppy iris syndrome in the literature to demonstrate this effect, or provide a model to test the impact of pharmacological agents.

### What this paper adds

- We have refined a mathematical model for floppy iris syndrome and provided a computer simulation to demonstrate the potential for iris displacement.
- Mechanical pupil expansion devices such as the Malyugin ring are very effective in preventing abnormal iris movement, even when modelling a floppy iris scenario with 40% degradation of its elastic properties.
- Altering the parameters for this model provides a proof for the impact of mechanical expansion devices in addressing abnormal iris behaviour in floppy iris syndrome, and can be used to test the impact of other adjuncts such as pharmacological agents.

## Supplementary information

Online supplementary video

SUPPLEMENTARY FIGURE A

SUPPLEMENTARY FIGURE B

Supplementary legends

## References

[1] Lockington D, Macdonald EC, Young D, Stewart P, Caslake M, Ramaesh K. Presence of free radicals in intracameral agents commonly used during cataract surgery. Br J Ophthalmol. 2010;94:1674–7. 10.1136/bjo.2009.171009 20644213

[2] Vazquez-Ferreiro P, Carrera-Hueso FJ, Barreiro-Rodriguez L, Diaz-Rey M, Poquet-Jornet JE, Ramón-Barrios MA, et al. Effectiveness of intracameral phenylephrine in achieving mydriasis and reducing complications during phacoemulsification: a systematic review and meta-analysis. J Ocul Pharmacol Ther. 2017. 10.1089/jop.2017.0084. 10.1089/jop.2017.0084 29099656

[3] Lay Suan AL, Hamzah JC, Ken TS, Mansurali VN. Intracameral mydriatics versus topical mydriatics in pupil dilation for phacoemulsification cataract surgery. J Cataract Refract Surg. 2017;43:1031–5. 10.1016/j.jcrs.2017.05.031 28917402

[4] Schulz CB, Goverdhan SV, Humphry RC. An evaluation of intracameral mydriasis for routine cataract surgery. Br J Ophthalmol. 2017. 10.1136/bjophthalmol-2017-310510. 10.1136/bjophthalmol-2017-310510 28903962

[5] Labetoulle M, Findl O, Malecaze F, Alió J, Cochener B, Lobo C (2016). Intracameral Mydrane Study 2 Group. Evaluation of the efficacy and safety of a standardised intracameral combination of mydriatics and anaesthetics for cataract surgery. Br J Ophthalmol.

[6] Behndig A, Korobelnik JF (2015). Mydriatic insert and intracameral injections compared with mydriatic eyedrops in cataract surgery: controlled studies. J Cataract Refract Surg.

[7] Tian JJ, Garcia GA, Karanjia R, Lu KL (2016). Comparison of 2 pupil expansion devices for small-pupil cataract surgery. J Cataract Refract Surg.

[8] Zarei-Ghanavati S, Bagherian H (2015). Stabilizing the capsular bag and expanding the pupil with a pupil expansion device. J Cataract Refract Surg.

[9] Wilczynski M, Wierzchowski T, Synder A, Omulecki W. Results of phacoemulsification with Malyugin ring in comparison with manual iris stretching with hooks in eyes with narrow pupil. Eur J Ophthalmol. 2013;23:196–201. 10.5301/ejo.5000204 23112041

[10] Roberts TV, Lawless M, Hodge C (2013). Laser-assisted cataract surgery following insertion of a pupil expander for management of complex cataract and small irregular pupil. J Cataract Refract Surg.

[11] Dick HB, Schultz T. Laser-assisted cataract surgery in small pupils using mechanical dilation devices. J Refract Surg. 2013;29:858–62. 10.3928/1081597x-20131115-06 24404609

[12] Malyugin B, Sobolev N, Arbisser LB, Anisimova N (2016). Combined use of an iris hook and pupil expansion ring for femtosecond laser-assisted cataract surgery in patients with cataracts complicated by insufficient mydriasis and an ectopic pupil. J Cataract Refract Surg.

[13] Malyugin BE. Recent advances in small pupil cataract surgery. Curr Opin Ophthalmol. 2017. 10.1097/ICU.0000000000000443. 10.1097/ICU.0000000000000443 29059105

[14] Malyugin B (2017). Cataract surgery in small pupils. Indian J Ophthalmol.

[15] Malyugin B. Small pupil phaco surgery: a new technique. Ann Ophthalmol. 2007;39:185–93. 10.1007/s12009-007-0023-8 18025623

[16] Chang DF, Campbell JR (2005). Intraoperative floppy iris syndrome associated with tamsulosin. J Cataract Refract Surg.

[17] Chang DF (2008). Use of Malyugin pupil expansion device for intraoperative floppy-iris syndrome: results in 30 consecutive cases. J Cataract Refract Surg.

[18] Guthrie S, Jensen T, Hartley RC, Ramaesh K, Lockington D. Assessing the accuracy of intracameral phenylephrine preparation in cataract surgery. Eye. 2018. 10.1038/s41433-018-0143-y. 10.1038/s41433-018-0143-y PMC6189205 29907787

[19] Lockington D, Luo X, Wang H, Hill NA, Ramaesh K (2012). Mathematical and computer simulation modelling of intracameral forces causing pupil block due to air bubble use in Descemet’s stripping endothelial keratoplasty: the mechanics of iris buckling. Clin Exp Ophthalmol.

[20] Qi N, Lockington D, Wang HM, Cai L, Ramash K, Luo XY. Modelling floppy iris syndrome and the impact of phenylephrine on iris buckling. Int J Appl Mech. 2018;10:1850048-1-13.

[21] Canning CR, Greaney MJ, Dewynne JN, Fitt AD (2002). Fluid flow in the anterior chamber of a human eye. Math Med Biol.

[22] Fitt AD, Gonzalez G (2006). Fluid mechanics of the human eye: aqueous humour flow in the anterior chamber. Bull Math Biol.

[23] Huang EC, Barocas VH (2004). Active iris mechanics and pupillary block: steady-state analysis and comparison with anatomical risk factors. Ann Biomed Eng.

[24] Huang EC, Barocas VH (2006). Accommodative microfluctuations and iris contour. J Vis.

[25] Mapstone R (1970). Forces determining pupil size. Exp Eye Res.

[26] Miller K (2005). Method of testing very soft biological tissues in compression. J Biomech.

[27] Heys JJ, Barocas VH, Taravella MJ (2001). Modeling passive mechanical interaction between aqueous humor and iris. J Biomech Eng.

[28] Heys JJ, Barocas VH (1999). Mechanical characterization of the bovine iris. J Biomech.

[29] Whitcomb JE, Barnett VA, Olsen TW, Barocas VH (2009). Ex vivo porcine iris stiffening due to drug stimulation. Exp Eye Res.

[30] Tan RKY, Wang X, Perera SA, Girard MJA (2018). Numerical stress analysis of the iris tissue induced by pupil expansion: comparison of commercial devices. PLoS ONE.

[31] Invernizzi A, Cigada M, Savoldi L, Cavuto S, Fontana L, Cimino L (2014). In vivo analysis of the iris thickness by spectral domain optical coherence tomography. Br J Ophthalmol.

