# Supplementary figures and images for: Modelling floppy iris syndrome and the impact of pupil size and ring devices on iris displacement
Source: Eye (Lond). 2020 Feb 4;34(12):2227–34. doi: 10.1038/s41433-020-0782-7 (PMC7784872; doi:10.1038/s41433-020-0782-7)

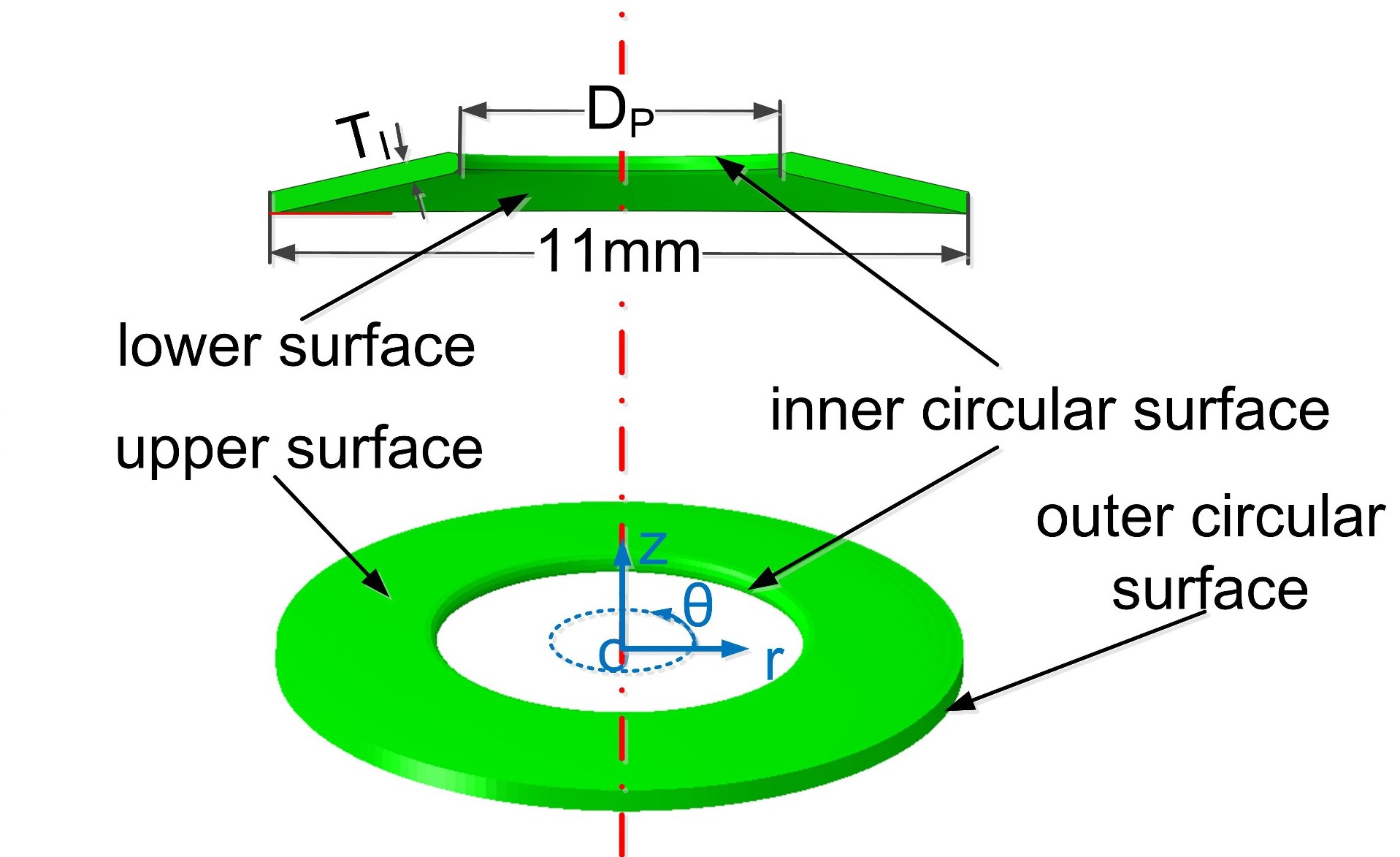

Supplement: Supplementary file 2 — SUPPLEMENTARY FIGURE A [file 41433_2020_782_MOESM2_ESM.jpg]

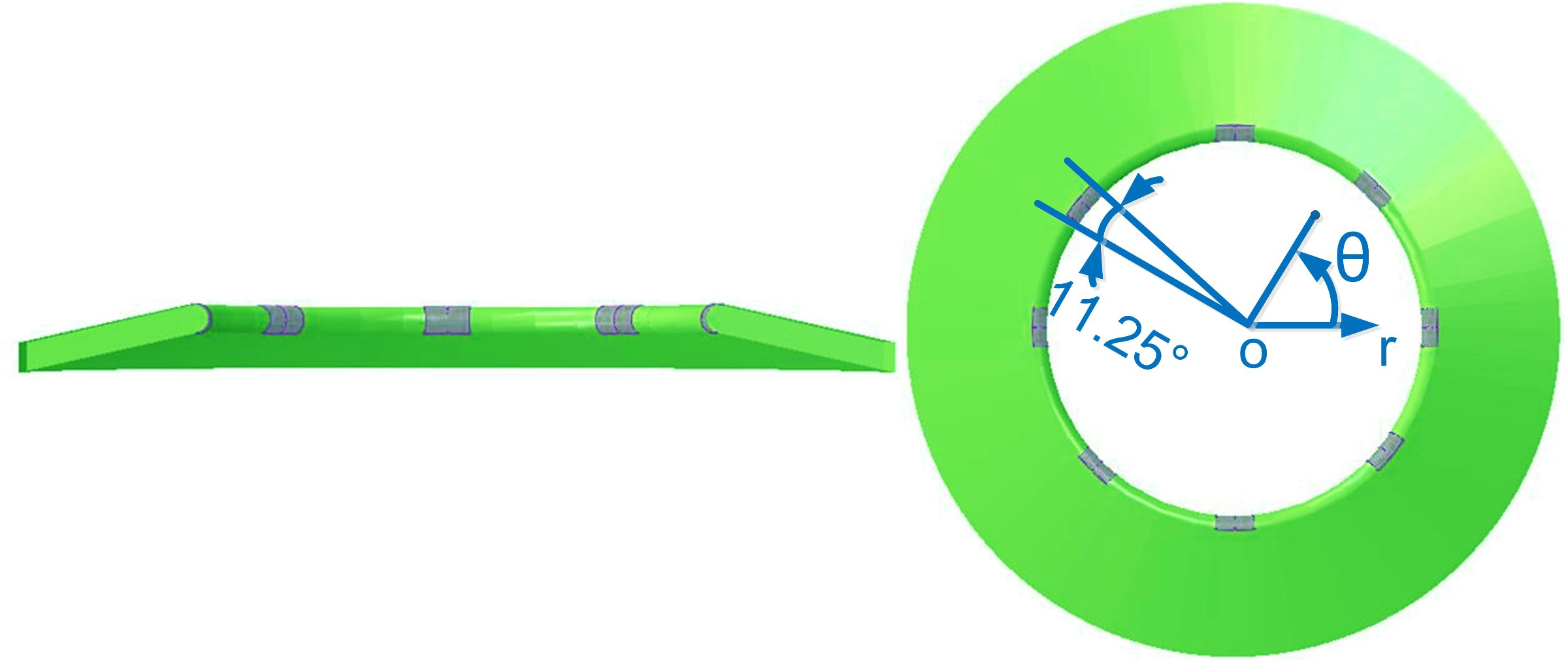

Supplement: Supplementary file 3 — SUPPLEMENTARY FIGURE B [file 41433_2020_782_MOESM3_ESM.png]
