# Supplementary material for: Modelling floppy iris syndrome and the impact of pupil size and ring devices on iris displacement
Source: Eye (Lond). 2020 Feb 4;34(12):2227–34. doi: 10.1038/s41433-020-0782-7 (PMC7784872; doi:10.1038/s41433-020-0782-7)
Supplement: Supplementary file 4 — Supplementary legends [file 41433_2020_782_MOESM4_ESM.docx]

**SUPPLEMENTARY FIGURE A**. Illustration of basic model of geometric parameters for iris, where D_P_ is the diameter of pupil; T_I_ is the thickness of the iris; with the local cylindrical coordinate system.

**SUPPLEMENTARY FIGURE B.** Illustration of basic iris model with Malyugin ring parameters - Eight uniformly distributed inner pinned areas (grey areas) of inner iris surface, where each area covers 11.25 degrees.

**Supplementary online video.** Computer simulation demonstrating the difference in iris buckling behaviour of floppy iris syndrome compared to the reduction in iris movement despite higher critical pressures due to the 7mm Malyugin ring parameters exerting mechanical restriction.
